# Influence of Si_3_N_4_ fillers and pyrolysis profile on the microstructure of additively manufactured silicon carbonitride ceramics derived from polyvinylsilazane

**DOI:** 10.1080/14686996.2024.2363170

**Published:** 2024-06-06

**Authors:** Alper Balkan, Xifan Wang, Aleksander Gurlo

**Affiliations:** a Faculty III Process Sciences, Institute of Materials Science and Technology, Chair of Advanced Ceramic Materials, Technische Universität Berlin, Berlin, Germany; b Laboratory for Processing of Advanced Composites (LPAC), Ecole Polytechnique Fédérale de Lausanne (EPFL), Lausanne, Switzerland

**Keywords:** Polymer-derived ceramics, preceramic polymers, polyvinylsilazane, silicon nitride, digital light processing

## Abstract

In this work, various methods were used to improve the printability of a photocurable polyvinylsilazane resin filled with silicon nitride particles for digital light processing. The developed resin was used as a preceramic polymer for polymer-to-ceramic conversion. The pyrolysis-induced structural changes of the additively manufactured objects were evaluated by comparing samples with different thicknesses, filler amounts and heating profiles. The printed green body retained its original geometry better and showed fewer cracks due to the addition of silicon nitride particles to the resin. Based on the thermally induced changes in a polyvinylsilazane resin system, a customized heating profile for the pyrolysis process was developed, which contributed to the reduction of pores and cracks while the average pyrolysis heating rate remained relatively high. This work provides insight into the pyrolysis of additively manufactured preceramic polymer green bodies and highlights various strategies for additive manufacturing of polymer-derived ceramics.

## IMPACT STATEMENT

The presented work systematically demonstrates the microstructural optimization of additively manufactured polymer-derived ceramics through combination of high refractive index filler inclusion and pyrolysis procedure customization.

## Graphical abstract

**Figure:**
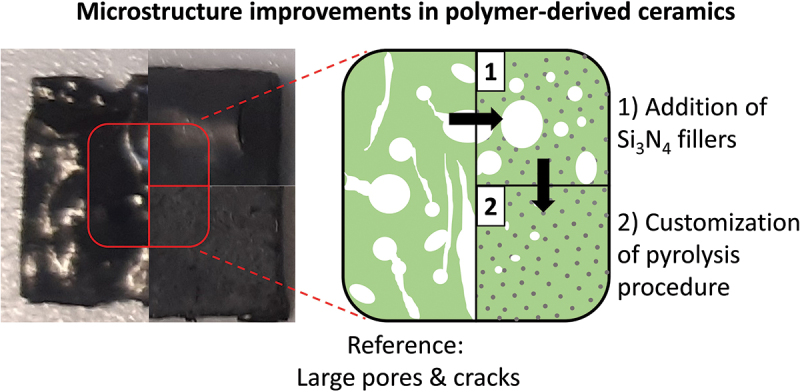

## Introduction

Polymer-derived ceramics (PDCs) are an extensively studied class of ceramic materials that are synthesized by pyrolyzing preceramic polymers. For example, silicon carbides, oxycarbides, nitrides, carbonitrides are obtained by cross-linking and pyrolysis of Si-containing polymers such as polycarbosilanes, polycarbosiloxanes, polysilazanes and polysilylcarbodiimides [1]. The PDCs demonstrate attractive thermomechanical [2], electromagnetic [3], optical [4] properties, biocompatibility [5] as well as adjustable functionality [6] attributed to the customizable chemical composition and unique nanostructure [7]. Pyrolysis usually takes place under an inert atmosphere and at relatively low temperatures (~1000°C), making it economically beneficial compared to conventional sintering temperature of ceramic powders [8]. Moreover, various polymer forming methods can be applied and integrated with the cross-linking mechanisms of preceramic polymer, allowing them to be shaped into components. This has been particularly useful for their direct application in stereolithography (SLA), a three-dimensional (3D) printing process utilizing a moving laser, or light projection through digital light processing (DLP) chips to construct 3D objects in a layer-wise manner with liquid photopolymers. The PDC route provides a promising alternative to the slurry-based ceramic stereolithography (cerSLA) and in general the additive manufacturing (AM) of ceramics, hence engaging noteworthy attentions from academy and industry fields.

The highly praised features of preceramic polymer stereolithography such as the well-preserved shape after pyrolysis and the predictable shrinkage of the additively manufactured objects has recently been demonstrated for a range of the PDCs [9]. PDCs of various compositions including silicon carbonitride (SiCN) [10,11], silicon oxycarbide (SiOC) [9,12,13] and silicon borocarbonitride (SiBCN) [14] were fabricated by additive manufacturing of the green bodies via stereolithographic approach. Many of the PDC related studies involve (meth)acrylates to build a strong network via illumination in a reasonably short amount of time. However, these types of resins can suffer from considerably high shrinkage, internal stress and warpage development during the photopolymerization [15,16]. Nevertheless, avoiding (meth)acrylates for both PDC and ceramic particle-based DLP printing is possible by methods like thiol-ene click chemistry. The stereolithographical AM of PDCs can grant even higher detail products if the light-induced cross-linking is achieved by thiol-ene click chemistry owing to more homogeneous and less strained step-growth network formation. This photopolymerization route can be considered favorable due to its oxygen resistance [17], high efficiency [18], low shrinkage stress [19,20] and low warpage for AM [21]. The thiol-ene click chemistry, although its use is still uncommon for additively manufactured PDCs, was utilized to create SiOC lattices previously [9]. Furthermore, our group has recently developed a versatile AM method to produce high compressive strength-to-weight ratio SiOC lattices and macrostructures, which was also used to prepare SiC(O) and SiCN(O) objects using a simple benchtop DLP printing setup and thiol-ene click chemistry [22]. In the mentioned study, vinyl containing preceramic oligomers were photocross-linked with difunctional thiols to form complex objects by DLP. The changes in the structure were examined during as well as after the pyrolysis, which showed the nearly fully dense, defect-free structure of these PDCs. So far, the concept of ceramics AM via stereolithography of preceramic polymers, followed by polymer to ceramic conversion through pyrolysis is proven feasible. The attainable geometry of the PDCs parts, nonetheless, is prescribed to lattices with thin struts less than 1 mm [23] or in extreme cases, to micro-3D structures below 100 micrometers [24].

The success of the PDC product, or specifically the flawless conversion of preceramic polymer part to dense ceramic, is contingent upon its behavior amid the thermal treatment. The PDC is formed from preceramic polymers starting with the cross-linking (<400°C) and continued with the cleavage and reordering of chemical bonds, causing gas release and open pore network generation, as stated previously [25]. Afterwards, the material starts to shrink due to the densification. The simultaneous gas evolution and volumetric shrinkage (that corresponds typically to 20–30% linear shrinkage) can result in micro- and macro-scale pores and cracks in the ceramics [7]. Such structural inhomogeneities reduce the ceramic density and pose risks to bulky objects, although lattice structures with thin cell walls are less prone to fail, as the diffusion length for outgas is sufficiently short. It was demonstrated [26] that the fracture occurring in bulky SiOC ceramics can be associated with the final gas release from the structure between 750°C and 800°C, which comprises largely hydrogen and methane. During that stage, the PDC structure hardens due to ceramization, hence is unable to shrink via viscous flow (diminishes at ~1000°C), elevating the possibility of fracture when high heating rates or thick geometries are employed. This is due to the mechanical stresses stemming from the conversion gradient between the exterior and interior regions of the object. Although utilizing high pressure of the aforementioned gases can be helpful to avoid fracture, it also decelerates the removal of these gas species significantly [26,27]. Careful choosing of the heating rate is another vital aspect of successful pyrolysis, especially for thicker samples [26]. As an example, Yang et al. demonstrated that dense and crack-free SiOC ceramics can be prepared at the cost of employing slow heating rates for the pyrolysis [28]. However, employing slow heating rates leads to prolonged ceramization durations. Thus, alternative solutions are required if long pyrolysis durations are to be avoided.

Inorganic particles such as silicon nitride (Si_3_N_4_) or silicon carbide (SiC) can be introduced to the preceramic polymer as inert fillers to effectively reduce pores and cracks while creating composite materials. They mainly serve the purpose of reducing the volumetric shrinkage of PDC component by being inert, and providing escape paths for emerging gases created during pyrolysis [29], although the cause for those means of escape remains unproven. Incorporation of these fillers can also improve the mechanical properties of obtained PDC beginning from low loadings (<10 vol.%) and reach zenith around 40–50 vol.% before losing the additional strength attributed to high porosity formation [30]. Nevertheless, adding particles in photocurable preceramic polymer resin causes challenges for the stereolithography process by significantly reducing resin’s cure depth [31], especially for particles with heavy absorption near the ultraviolet (UV) wavelengths used for polymerization. The low cure depth can also make the printing of geometries with fine details less feasible. Surface modification and intentional oxidation of the particles to change the refractive index and particle size control were demonstrated as efficient methods to adjust the cure depth [32–34] as well as viscosity [34]. There have been some efforts to produce particle-filled SiCN (and derivatives) PDCs with stereolithographic methods. An example was the making of Si_3_N_4_ whiskers (60 wt.% of polymer) – filled SiBCN objects with reduced shrinkage along with enhanced strength and ceramic yield [35] without generating microcracks. In another work, silica nanoparticles (10 wt.%) were used to increase the elastic modulus and Vickers hardness of a SiCN matrix, so to produce a microreactor [36]. Furthermore, another SiCN ceramic matrix composite (CMC) was prepared with both silica nanoparticles (20 wt.%) and SiC nanofibers (1 wt.%) as fillers. The strategy again increases the ceramic yield and leads to component with less porosity and increased mechanical strength [37]. Further on, a better thermal stability and lower shrinkage for the composites in contrast to the PDC derived from pure preceramic polymer were reported. However, a deep insight to how the addition of inert particles combined with customized pyrolysis profile facilitates the conversion of additively manufactured preceramic polymer to ceramics along with influence of resultant microstructures on the mechanical properties is still lacking.

In this work, crack-free SiCN ceramic objects are fabricated using a photocurable Si_3_N_4_-filled polyvinylsilazane resin in a benchtop DLP printer via thiol-ene click chemistry. Through optimization of the slurry composition, printing parameters and pyrolysis profile, additively manufactured preceramic polymer cuboids are successfully converted to SiCN/Si_3_N_4_ nanocomposites with reduced volumetric shrinkage and impeded crack formation as well as increased ceramic yield. This study systematically shows the advantages of adding Si_3_N_4_ fillers and utilizing a customized pyrolysis profile (according to the thermal behavior of the material) for additively manufactured PDC parts.

## Experimental

### Materials selection

A commercial polyvinylsilazane (PVSZ, Durazane 1800, durXtreme GmbH, Germany) with a molecular structure of [(C_2_H_3_)(CH_3_)Si-NH]0.2_n_[H(CH_3_)Si-NH]0.8_n_ [38] was used as the preceramic polymer for all resins together with the cross-linker 1,6-hexanedithiol (HDT, 98%, J&K Scientific GmbH, Germany). The phenylbis(2,4,6-trimethylbenzoyl)phosphine oxide (BAPO: bisacylphosphine oxide, 97%, Sigma-Aldrich, Germany) was used as Norrish type I photoinitiator. Furthermore, Sudan Orange G (SOG, 85%, Sigma-Aldrich, Germany) was utilized as photoabsorbers. To prolong the shelf life of resin, hydroquinone (HQ, ≥99%, Sigma-Aldrich, Germany) was selected as a radical scavenger. The radical scavenger and photoabsorbers were delivered as mixture dissolved in tetrahydrofuran (THF, ≥99%, Sigma-Aldrich, Germany). O-(2-aminopropyl)-O’-(2-methoxyethyl)-propylene glycol (APMG, Sigma-Aldrich, Germany) was used as the dispersant of the Si_3_N_4_ particles in non-aqueous medium [39]. In this work, two types of Si_3_N_4_ particles were selected. One is a grey α-Si_3_N_4_ powder (HeFei Morke Advanced Material Technology Co., China) and the other one is an α-Si_3_N_4_ (LC12N, H. C. Starck, Germany) powder with a whitish appearance. These predominantly α phase powders are referred as the Grey Si_3_N_4_ the White Si_3_N_4_ respectively throughout this article. The chemicals were used as purchased without further purification.

### Resin preparation

The resin preparation started with dissolving SOG, HQ, APMG in PVSZ by magnetic stirring. The respective amounts of resin ingredients can be found in Table 1. If a significant amount of THF (>0.5 mL for a total of 20 mL resin) was used during preparation, this step was followed with drying under vacuum at room temperature to eliminate THF. Afterwards, HDT and BAPO were added until a clear preceramic polymer solution was obtained, which is further stored in dark to avoid undesired polymerization. In order to prepare the slurry, Si_3_N_4_ powders were first dried for an hour at 90°C, and then mixed with the resin. The slurry was homogenized with planetary ball milling (PM4, Retsch GmbH, Germany) for 10 min followed by magnetic stirring. For the resins of films cured with UV illumination source, the photoinitiator 2,2-dimethoxy-2-phenyl acetophenone (DMPA) was used instead of BAPO.

**Table 1.** Resin compositions and printing parameters.

Resins	Wt._HDT_ /Wt._PVSZ_	BAPO (wt.%)*	SOG (wt.%)*	APMG (wt.%)*	HQ (wt.%)*	Powder	Powder Amount (vol.%)**	Layer Thickness (μm)	Exposure Time (s)
Resin 1	0.24	1	0.2		~0.04			30	2.5
Resin 2	0.24	0.5	0.15	5	~0.04	White Si_3_N_4_	5	30	12
Resin 3	0.24	0.5	0.15	5	~0.04	White Si_3_N_4_	10	30	30–40

*wt.% with respect to the total weight of the liquid phase of resin.

**vol.% with respect to the entire resin.

### Cure depth measurements and additive manufacturing

**Figure 1.**
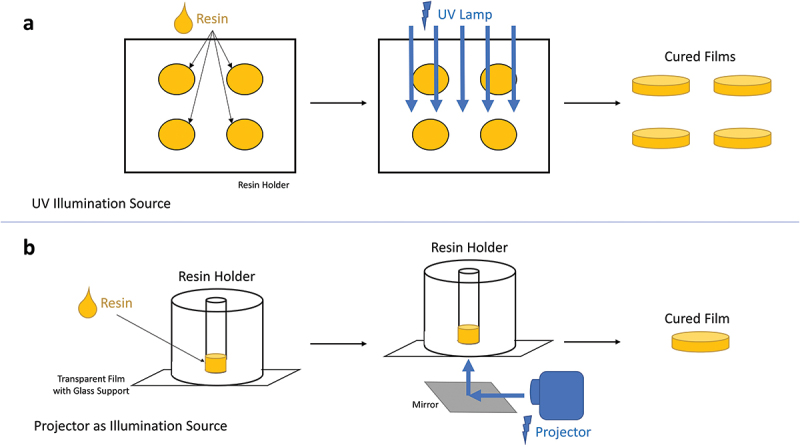
Cure depth experiment setups: (a) with UV illumination source, (b) with projector.

An open-source DLP printer, LittleRP2 (Brad Hill, Santa Barbara, CA, U.S.A.), was utilized for the cure depth measurements and additive manufacturing. The printer was coupled with an Acer X152H projector (New Taipei City, Taiwan) as the illumination source. The controlling of the printing equipment as well as the slicing of the CAD files were done using the software Creation Workshop (DataTree3D, Dallas, TX, U.S.A.). Furthermore, a UV illumination source (UV-Belichtungsgerät 1, isel, proMA Technologie GmbH) was used for the cure depth experiments involving the photoinitiator DMPA. The films from cure depth experiments (Figure 1) were measured with a caliper, and the values were plotted to create the Jacobs working curves [40].

**Figure 2.**
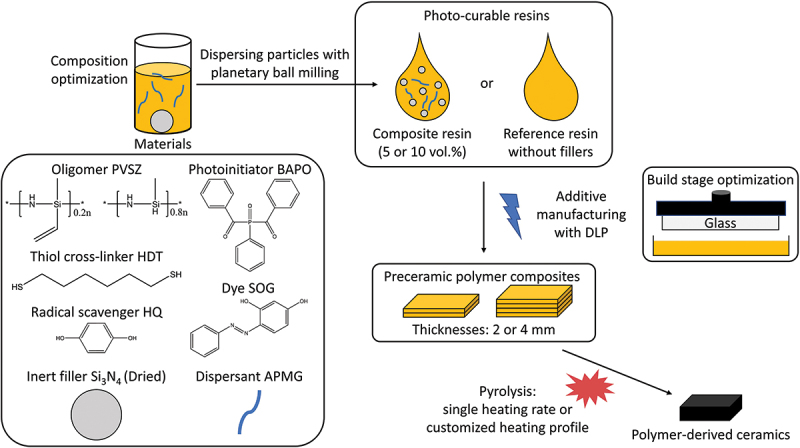
Schematic showing the parameters employed for preparing the pyrolyzed 3D objects.

For the additive manufacturing, 30 μm was chosen as the layer thickness. The layers were cured for 2.5, 12–20 and 30–40 s for the powder-free resin, 5 vol.% Si_3_N_4_-loaded resins and the 10 vol.% Si_3_N_4_-loaded resins, respectively. Table 1 shows the main components and printing parameters of the three resins used for additive manufacturing of the objects for studying pyrolysis behavior. In addition, Figure 2 displays a schematic of preparing the various additively manufactured PDCs in this work.

### Pyrolysis profile

**Figure 3.**
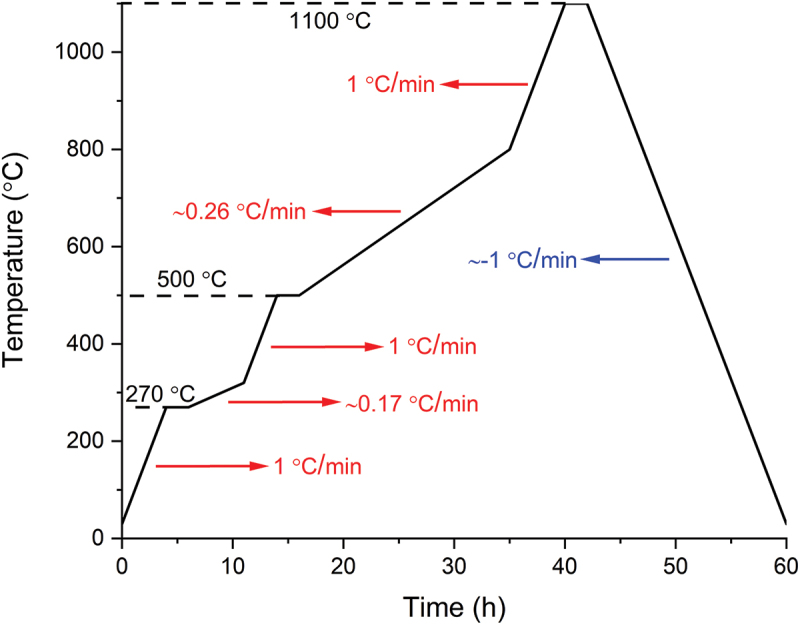
The customized heating profile with specific ramp rates and dwell temperatures.

The thermal treatment of fabricated objects was carried out in a tubular furnace (RE 2.1, Heraeus, Germany) under flowing nitrogen as protecting atmosphere. Two heating profiles were selected for comparison. The first heating profile possessed a single heating rate of 1°C/min from room temperature up to the target temperature of 1100°C. After 2 h of dwelling at 1100°C, the cooling was done at 1°C/min rate. The second heating rate was customized considering the thermogravimetric behavior of the PVSZ and allowing longer time at intervals of high structural change. This heating profile can be seen in Figure 3 and was only applied with 10 vol.% Si_3_N_4_-loaded square prisms.

### Characterization methods

The size distribution of Si_3_N_4_ powders was characterized by a laser diffraction particle size analyzer with wet measurement unit (LS 13 320, Beckman Coulter, CA, U.S.A.). The sample preparation was done by adding powder to a 100 mL beaker filled with 50 mL water, which was agitated with ultrasonication (Branson Digital Sonifier 450, U.S.A., 20 kHz, 80 Watt) at power setting 6 for 30 s at 70% efficiency. For the analysis Fraunhofer model and polarization intensity differential scattering technology (Patented by the company) was used with a size range of 0.04–2000 μm. Dynamic viscosity measurements of resins were done with a viscometer (Anton Paar MCR 301, Anton Paar GmbH, Graz, Austria) in plate–plate mode at 20°C for the shear rates between 0.1 and 100 s^−1^. The UV absorption of Si_3_N_4_ powders was assessed with a UV–VIS spectrometer (Lambda 900, PerkinElmer, Inc., MA, U.S.A.) in transmittance mode. The values were normalized according to the highest data point available. Thermogravimetry (TG) and differential thermal analysis (DTA) were conducted with an alumina reference in nitrogen atmosphere with STA 409 PC LUXX (Netzsch, Germany) from 25 to 1100°C with 5°C/min ramp rate. Mass spectrometry (MS) of the gas phase released during the thermal analysis was conducted with multiple ion detection by OMNi Star GSD 320 (Pfeiffer Vacuum, Germany). The skeletal densities of the pyrolyzed objects were measured with Archimedes method (Sartorius YDK 01, Sartorius AG, Germany) and calculated according to an ASTM standard [41]. The phase composition of the pyrolyzed 3D printed objects was analyzed with X-ray diffraction (XRD) (D8, Bruker Corporation, MA, U.S.A.) using CuKα radiation (1.5406 Å) in Bragg-Brentano geometry with the step size of 0.02° 2θ. The data analysis was performed with Diffrac-Plus/EVA software (Bruker AXS GmbH, Germany). The microstructure of the pyrolyzed objects was evaluated with radiography and micro-computed tomography (µCT) employing a custom-built setup equipped with a ~ 120×120 mm^2^ flat panel detector (Hamamatsu, Japan) and an air-cooled microfocus X-Ray source (Hamamatsu, Japan) at 100 kV/μA with the pixel pitch of the detector of 50 μm [42]. The radiography images were obtained with 7.5 magnification and an end pixel size of 6.67 μm. The µCT was done at 6.5 magnification with an end voxel size of 7.7 μm. The reconstruction of the tomogram was achieved using 1000 projections over 360 degrees (Octopus 8.8, Inside Matters, Belgium). Avizo 9 (FEI, U.S.A.) was employed for the 3D rendering and the porosity determination. For the scanning electron microscopy (SEM), the additively manufactured objects were ground, polished and coated with carbon. SEM images and elemental mapping (C, N, O, S, Si) were collected using 10 kV acceleration voltage with Gemini Leo 1530 FEG-SEM (Carl Zeiss Microscopy GmbH, Germany) and energy-dispersive X-Ray spectroscopy (EDX) system of Thermo Fisher Scientific.

## Results & discussion

### Influence of Si_3_N_4_ particles on the resin photopolymerization behavior

**Figure 4.**
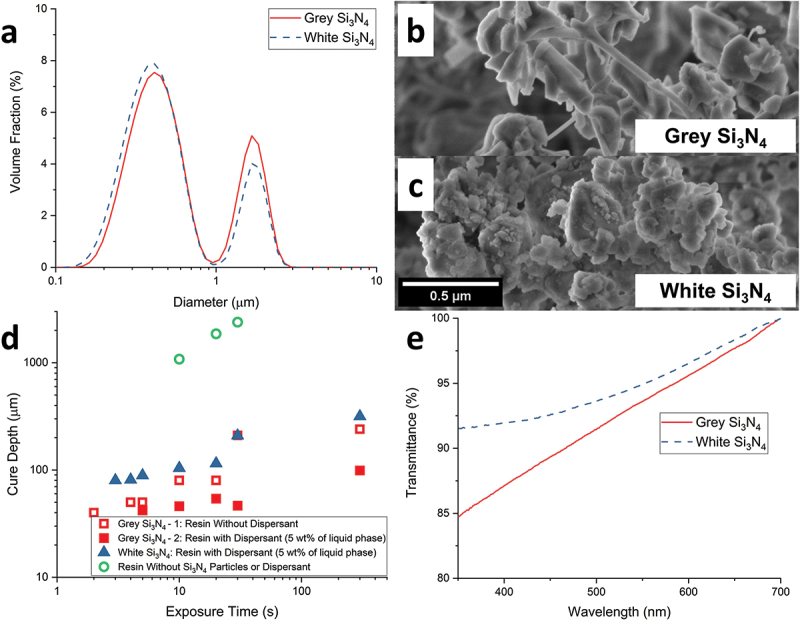
(a) The particle size distributions of Grey and White Si_3_N_4_ powders; (b, c) SEM images of Grey and White Si_3_N_4_ powders respectively; (d) cure depth against exposure time for resins with or without fillers and dispersant; (e) Visible transmittance spectra of Grey and White Si_3_N_4_ powders.

In order to integrate the ceramic particles in resins for the DLP printing of PDCs, the photo-curing behavior of resin was characterized first. The two Si_3_N_4_ powders used in this study, named as Grey and White Si_3_N_4_, were chosen based on their alike particle size distribution and dissimilar color. The particle size and size distribution determine the median particle size (d_50_), whereas the color of the powders is related to the refractive indices as well as the impurities present in these powders. These factors influence the cure depth together with the wavelength of the irradiation source and the interparticle spacing [43]. The laser diffraction particle size analyzer (Figure 4(a)) revealed the median particle size of Grey Si_3_N_4_ powder as 0.493 ± 0.005 μm, making it slightly larger than the White Si_3_N_4_ (0.457 ± 0.003 μm). Furthermore, their particle size distributions were both bimodal with two peaks around 0.4 and 1.65 μm. Nevertheless, silicon nitride powders may have sub-200 nm crystallites which can form strongly bound primary agglomerates with sizes 500–1000 nm [44]. Such crystallites can be difficult to detect with particle size analyzers, hence SEM images were obtained. The SEM images of both powders (Figure 4(b,c)) revealed that the second peaks of both powders (~1.7 μm) were a direct sign of agglomeration as no comparable-sized particle was present. Moreover, the first peaks (~0.4 μm) comprised of both particles and primary agglomerates, which was attributed to the presence of closely located sub-100 nm crystallites. Thus, the effective particle size of Si_3_N_4_ in a resin is heavily contingent on the medium and the availability of possible dispersants.

Following the particle size characterization, the effect of particles on cure depths was examined. The first cure depth study was done with the photoinitiator DMPA and a UV source to demonstrate the cure depth of pure preceramic polymer resin. Without powder particles, the resin with 1 wt.% DMPA has 1080 μm cure thickness at 10 s and the cure depth reaches 2390 μm at the end of 30 s (Figure 4(d)). Remaining cure depth investigations were focused on involvement of the dispersant APMG and the difference between the Grey and White Si_3_N_4_. For that, the photoinitiator BAPO and the DLP projector light were used. As seen, the overall decrease in the film thickness shows the effect of absorption and reflection of powder particles on cure depth. The curing of a resin containing 10 vol.% Grey Si_3_N_4_ without the dispersant was possible within 2 s and at the end of 5 min, the measured thickness was 240 μm. Although the sample formed in 2 s lacked mechanical stability and a wrinkled texture was formed to compensate as well as inflating the measured thickness (Figure S1), which can be detrimental for a 3D printing layer. When the resin with dispersant was used, the cure depth further decreased to around 100 μm, which could be due to the breaking of agglomerates and a much lower effective particle size. Moreover, the three samples with the least exposure times (5, 10, 20 s) also suffered from wrinkled texture, making the fourth data point (30 s) of set Grey Si_3_N_4_ − 4 less than the third (20 s). Finally, using White Si_3_N_4_ instead of Grey Si_3_N_4_ leads to a less reduction of cure depth, which is in agreement with a recent study [45]. This result can be justified practically by analyzing the UV–VIS absorption spectra of the two powders dispersed in ethanol (Figure 4(e)) in addition to the theoretical explanation mentioned earlier. It can be observed that the transmittance of the White Si_3_N_4_ powder is comparatively higher than the Grey Si_3_N_4_ powder, especially at shorter wavelengths.

The effect of powders on resin viscosity was further investigated to determine the resin’s applicability with the DLP printer. In Figure S2, the dynamic viscosity profiles of PVSZ resins with varying amounts of powder are presented. The PVSZ displayed Newtonian-like fluid behavior and had 43 mPa.s viscosity at 1.12 s^−1^ shear rate (Table S2). Resin 1, the resin without powder particles, demonstrated a slightly lower viscosity due to the presence of the crosslinker HDT as reactive diluent. The shear thinning behavior seen with Resin 1 was associated with the PVSZ molecules, which could already be forming a polymer network in air [46]. Furthermore, the resins with ceramic fillers, Resin 2 and Resin 3, showed clear shear thinning behavior due to the incorporated ceramic particles. Besides, all resins have relatively low viscosities due to low particle loading, hence they were easily processable with DLP printing.

### DLP printing of PVSZ resins filled with Si_3_N_4_ particles

To study the thermal pyrolysis behavior, square prisms with dimensions of 15 × 15x2 and 15 × 15x4 mm^3^ (LxWxT) were fabricated. Based on our previous experience with the preparation of photocurable preceramic polymers [22], within Resin 1 HDT and PVSZ were mixed with stoichiometrically equal amounts of functional groups in order to sustain high cross-link density. After the introduction of ceramic particles, the resin’s cure depth largely reduced and led to severe printing challenges, as freshly polymerized layer adhered poorly to the previous printing layer. Consequently, parts often detached from the build plate and got stuck at the vat bottom during the printing process. Hence, adjustments were made to prepare Resin 2 and 3 including adjusting the dye and the photoinitiator concentrations to 0.15 and 0.5 wt.%, respectively, to prevent overcuring while maintaining sufficient cure depth. Moreover, exposure time was increased from 12–20 to 30–40 s when the particle loading was raised from 5 vol.% to 10 vol.%. Furthermore, build stage materials were optimized to improve the adhesion of the printed samples. Among aluminum, sand-blasted aluminum, steel, glass and a build stage coated with a thin layer of Resin 1, build stage out of glass was found to provide better adhesion and more stable prints empirically. With the above-mentioned improvements, finally polysilazane resin filled with Si_3_N_4_ particles could be used in DLP printing to fabricate square prisms as case study for thermal pyrolysis behavior.

### Polymer to ceramic transformation and the microstructure investigation of DLP printed objects

**Figure 5.**
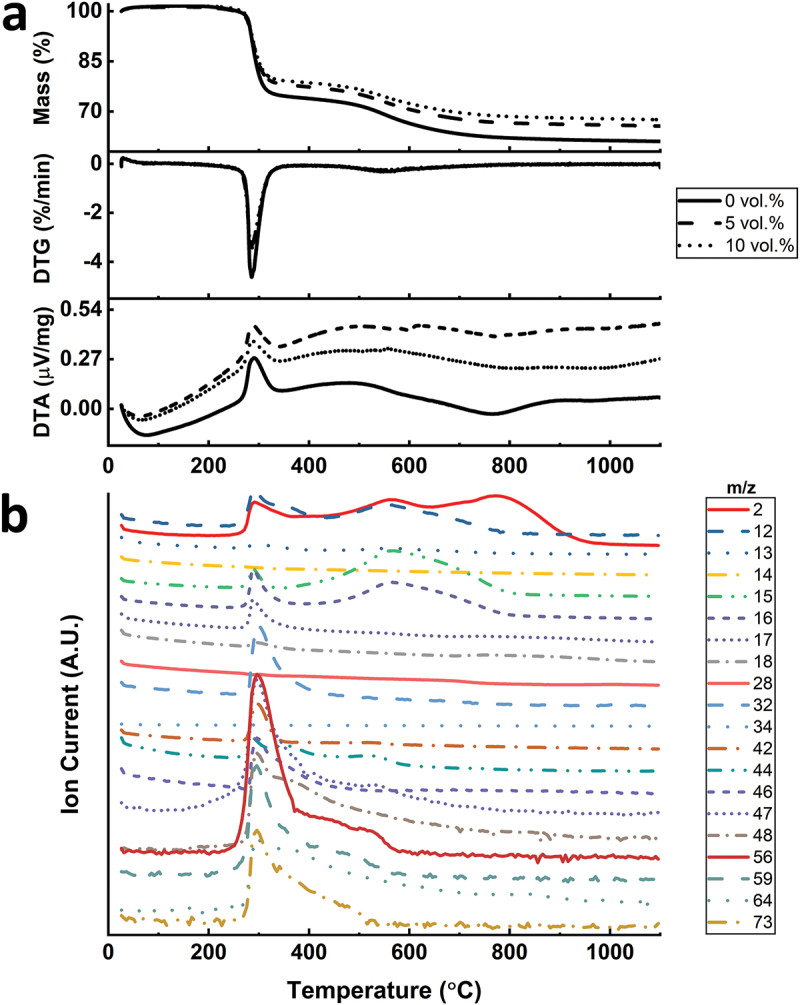
(a) DTG, TG and DTA graphs of samples with different Si_3_N_4_ filler loadings; (b) corresponding gas species released from the sample without Si_3_N_4_ fillers.

Simultaneous thermal analysis coupled with mass spectrometry was conducted to monitor the polymer-to-ceramic conversion of photo-cured objects under inert atmosphere (Figure 5). The thermogravimetric profiles of samples printed with three resins were similar in terms of the peak position and intensity. Samples prepared with higher amount of fillers resulted in reduced mass loss, as the ceramic yield increased from 61 wt.% to 66 wt.% and 68 wt.% by incorporating 5 vol.% and 10 vol.% fillers, respectively. The result was expected due to Si_3_N_4_ particles acting as inert fillers during the pyrolysis. The main mass loss, where mostly exothermic gas release, occurred around 300°C, starting at ~270°C and ending at ~340°C as shown in the differential thermogravimetry (DTG) curve (Figure 5(a)). This main mass loss account for between 20 and 25 wt.% in these samples. The pyrolyzed object further experienced a second mass loss due to ceramization beginning from 500°C up to 800°C. The overall endothermic structural change was mainly complete at ~900°C as suggested by the relative constancy of DTA graphs afterwards demonstrating the completion of the ceramization process at this temperature.

Mass spectrometry was used to analyze the released gases during pyrolysis (Figure 5(b)). The regions of pronounced gas emission lie affirmatively with the two mass loss stages identified in the TG curve. Around the major mass loss stage at 300°C, gases with molecular weights from 15 to 17 g/mol were identified, exhibiting the ammonia release, which could be due to the transamination reactions. The material could be further cross-linked during this stage [47]. The mineralization process, which can start at 450°C for polyvinylsilazanes, causes the cleavage of C-C, C-H, Si-C and N-H bonds [48]. This process causes additional gas release, which were noticed in the mass spectrometry between 496°C and 566°C. The released molecules at this region are mainly hydrogen (m/z = 2) and hydrocarbons that may also contain sulfur such as CH_4_, C_4_H_8_ and CH_3_SH (m/z = 12, 13, 15, 16, 44, 56). Above 800°C, only hydrogen (m/z = 2) was detected, which shows the loss of hydrogen as the final step leading to the amorphous SiCN [49]. Further information about analyzed compositions is provided in Table S3.

The pyrolyzed SiCN derived from pure PVSZ resin did not display any XRD reflections, which indicated the amorphous structure of this material; indeed, the crystallization of the SiCN to Si_3_N_4_ or SiC occurs at higher temperatures (>1200°C) [48]. In contrast, the PDC sample with 10 vol.% Si_3_N_4_ content showed low-intensity diffraction peaks attributable to the α- and β-Si_3_N_4_, i.e. fillers in the material (Figure S3).

**Figure 6.**
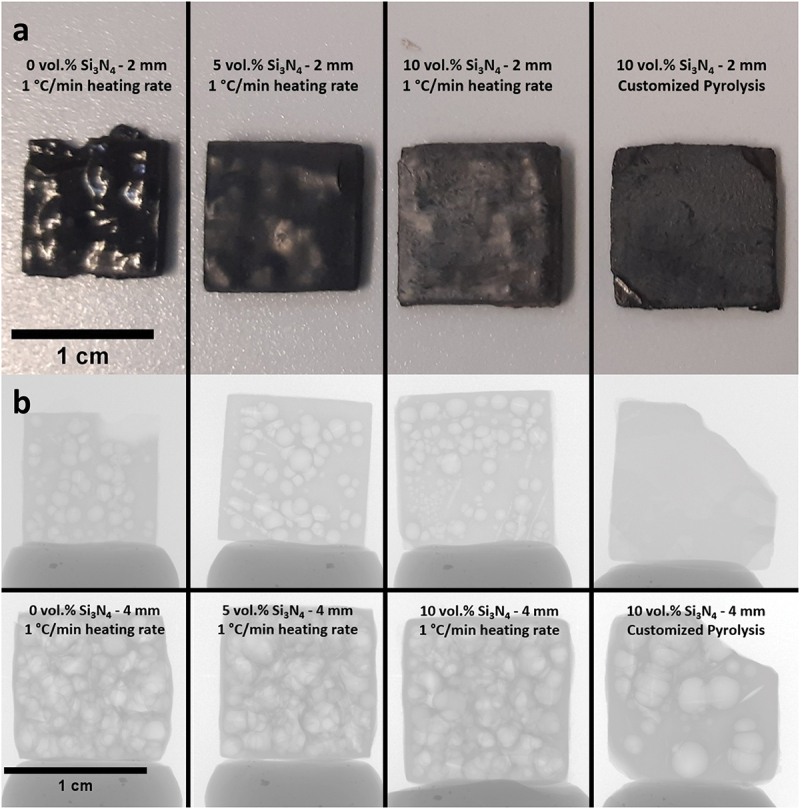
(a) 2 mm SiCN square prisms with 0, 5 and 10 vol.% Si_3_N_4_ loadings pyrolyzed at 1°C/min heating rate and the 2 mm 10 vol.% Si_3_N_4_-loaded square prism with customized pyrolysis profile; (b) the radiography images of pyrolyzed (1°C/min heating rate) SiCN samples with different Si_3_N_4_ loadings (0, 5, 10 vol.%) and thicknesses (2, 4 mm) as well as the 10 vol.% Si_3_N_4_-loaded samples with customized heating profile (2, 4 mm).

The printed square prisms with thicknesses of 2 and 4 mm were then pyrolyzed under nitrogen atmosphere to 1100°C either with 1°C/min heating rate or with the customized heating profile. As can be inferred from Table 2 and Figure 6(a), the introduction of fillers into the material notably reduced the shrinkage. The linear shrinkage fell from 35% (without filler) to 28% and 24% with 5 vol.% and 10 vol.% Si_3_N_4_ loaded samples, respectively, for the 2-mm-thick samples. The skeletal density of the 2 mm sample without fillers was 2.26 g/cm^3^ (Table 2), which is almost identical with a previously reported value of pyrolyzed Durazane 1800 [11]. The highest skeletal density (2.42 g/cm^3^) was observed with the 2 mm 10 vol.%-loaded sample with the custom heating profile. This was ascribed to both higher particle loading and the longer duration of the pyrolysis, leading to the timely evacuation of gases and a more successful densification during ceramization.

**Table 2.** Calculated linear shrinkage, mass loss, and skeletal density of pyrolyzed samples.

Samples	Thickness (mm)	Linear Shrinkage (%)	Mass Loss (%)	Skeletal Density (g/cm^3^)
Resin 1 (0 vol.%)	2	35	47.4	2.26
	4	25	45.9	2.19
Resin 2 (5 vol.%)	2	28	41.2	1.91
	4	25	41.2	1.91
Resin 3 (10 vol.%)	2	24	35.3	2.22
	4	22	35.8	2.22
Resin 3 (10 vol.%) with custom heating profile	2	24	35.5	2.42
	4	21	33.1	2.36

As demonstrated in the optical images (Figure 6(a)), the sample without Si_3_N_4_ fillers deformed severely by exhibiting macroscopic cracks and bumps owing to the violent gas release and simultaneous volumetric shrinkage that caused a significant amount of internal stress. Due to the increased diffusion length for outgas, the phenomenon was more evident with the 4-mm-thick samples by causing an artificially reduced linear shrinkage due to significant deformations in sample geometry. In contrast to the sample without fillers, square prisms fabricated with Si_3_N_4_ fillers showed fewer surface deformations and fractures, although not being able to eliminate them completely. It is suggested that the fillers reduced the shrinkage by staying inert upon ceramization and providing escape means to the outgas, therefore reduce the probability forming large defects [29]. Later, a custom pyrolysis profile (Figure 3) was applied to another set of 10 vol.% loaded samples, to decrease the ramp speed at temperatures of rapid structural change. This attempt was deemed successful as the comparison of the 2 mm thick 10 vol.% Si_3_N_4_-loaded prisms with and without the custom heating profile may suggest (Figure 6(a)). The prism pyrolyzed with the custom heating profile is completely flat without showing any distortions. The deformed corners were due to mishandling before pyrolysis.

The analysis of the microstructure was continued with radiography imaging (Figure 6(b)) to gain more insight about the structure and pores inside the materials. As seen in Figure 6(b), most specimens’ interior parts are filled with millimeter-sized, balloon-like pores, which can be considered as a visual representation of the accumulated volatile species during pyrolysis. Apparently, 4-mm-thick samples have more pores and larger pore size compared to their 2-mm-thick counterparts, demonstrating a clear positive correlation between the sample thickness and the presence of large defects. Notably, the 2-mm-thick sample pyrolyzed with custom heating profile, in which it is dwelled for 2 h at the beginning of both main outgassing stages and is followed with extremely low heating rate (<0.3°C/min), shows no sign of the high-volume sphere-like pores. It aligns with the specimen appearance that the surface is flat and smooth without cramping, exhibiting almost intact exterior and interior. Nonetheless, when the specimen thickness doubles, large spherical pores emerge again, however, in reduced quantity.

**Figure 7.**
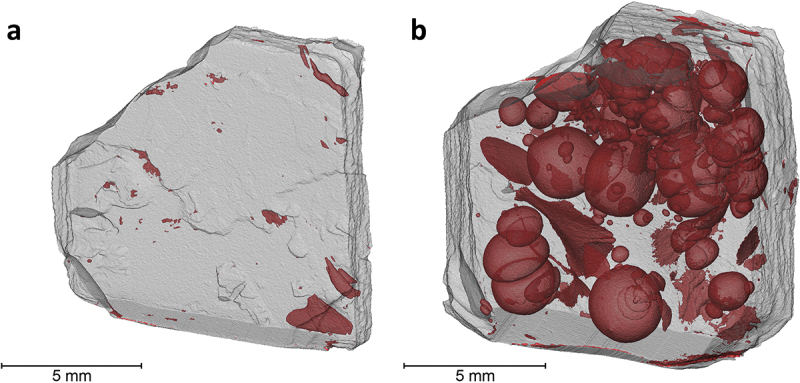
Micro-CT images of the 10 vol.% Si_3_N_4_-loaded Sic samples with customized heating profile for pyrolysis; (a) 2 mm, (b) 4 mm.

The 10 vol.% Si_3_N_4_-loaded SiCN samples with customized heating profile were further evaluated with µCT (Figure 7). The detection of pore sizes is limited with the voxel size of 7.7 μm. Bearing this in mind, the 2-mm-thick sample was found to have a remarkable 0.58% porosity, whereas the 4 mm sample had 22.17%. It is seen in Figure 7(a) that, using a customized pyrolysis profile allowed more time for structural stabilization in terms of allowing more gas diffusion out of the 2 mm object without causing massive, pyrolysis gas inflated spaces in the inner regions. However, the bulkiness of the printed objects with 4 mm thickness still managed to cause pore generation to a certain extent, which in a way demonstrates the limitation of the slower pyrolysis approach. Moreover, the shape of the pores could be non-spherical as well. The occurrence of this shape deviation was ascribed to the local availability of gas escape channels as well as their differing sizes. Such inhomogeneities could be the result of varying local concentration of Si_3_N_4_ particles and their sizes, as well as locally dissimilar polymer crosslink densities. Furthermore, as the shape deviations do not show any preferential orientation, as can be monitored in Figure 7(b). This observation can be used as an indication that the build orientation selected for printing does not have an apparent effect on pore evolution during pyrolysis.

**Figure 8.**
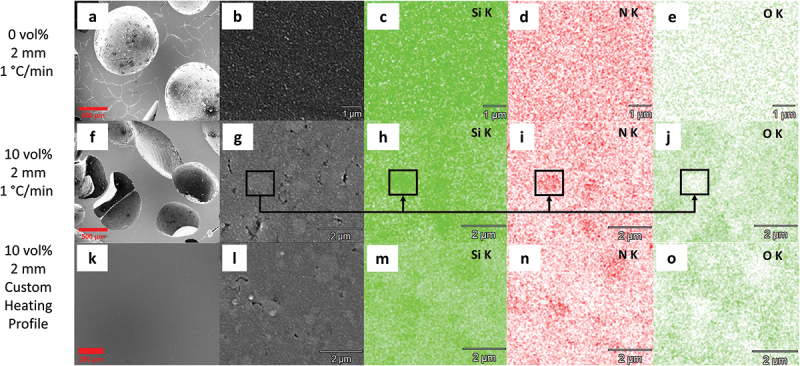
SEM images and EDX elemental mapping of SiCN samples: (a, b) SEM images of object prepared with 0 vol.% Si_3_N_4_ loading, 1°C/min heating rate and (c-e) elemental mapping of the object for Si (c), N (d), O (e) at b; (f, g) SEM images of object prepared with 10 vol.% Si_3_N_4_ loading, 1°C/min heating rate) and (h-j) elemental mapping of the object for Si (h), N (i), O (j) at g; (k, l) SEM images of object prepared with 10 vol.% Si_3_N_4_ loading, custom heating profile and (m-o) elemental mapping of the object for Si (m), N (n), O (o) at l.

SEM images and EDX element mapping of three 2-mm-thick samples were obtained to provide further information on the microstructure (Figure 8) regards the addition of Si_3_N_4_ fillers and customer pyrolysis profile. As seen in Figure 8(a), the 0 vol.% Si_3_N_4_-loaded sample (1°C/min heating rate) has not only millimeter-scaled spherical pores, but many interconnected cracks that encompass the whole material. It is reported that finding cracks and having a high porosity is common with quickly heated polymer derived SiCN ceramics as the gas release becomes more problematic at higher heating rates [50] as demonstrated by mass spectrometry at 5°C/min heating rate (Figure 5). As can be seen in Figures 8(b–e), the sample is element-wise homogeneous, and there was an emergence of sub-micron pores. The presence of these smaller pores not being able to stop millimeter-scale pores from occurring indicates that these sub-micron pores are not interconnected and are possibly generated during the hasty densification process. The EDX analysis revealed that oxygen and sulfur (Figure S4) are also present alongside silicon, carbon and nitrogen. This result agrees with the previous chemical composition studies where preceramic polymers are cross-linked via thiol-ene click chemistry [9,22].

It is shown in Figure 8(f) that adding 10 vol.% Si_3_N_4_ fillers, despite the ongoing presence of millimeter-scale pores, allowed vanishing of the interconnected cracks as solely spherical pores are left. In addition, its higher magnification SEM image (Figure 8(g)) reveals the presence of newly generated pores less than 100 nm, which are absent in the specimen prepared with only PVSZ. It is assumed that these new, third type of pores are generated by the incorporation of fillers. Indeed, previous studies [26,29] found out that the passive fillers serve mainly the purpose of reducing volumetric shrinkage and eliminating the presence of macro-scale defects like cracks and pores, possibly by particle-induced porosity and the short length of the polymeric material between particles. This claim is in accordance with the SEM investigation of this work, in which passive fillers mostly eliminated the macroscopic crack formation by inducing additional porosities, from which volatile species generated during pyrolysis can easily escape. In Figures 8(g–j), the local color differences further indicate the existence of a second material, the Si_3_N_4_ fillers. These light grey regions vary between a few hundred nanometers to micrometer-sized spots in accordance with the particle size distribution, demonstrating aggregates of the Si_3_N_4_ crystallites. The elemental contrast in the mapping of Si, N and O (Figures 8(h–j)) further verifies the light grey regions as Si_3_N_4_ fillers again, in which there is higher concentration of silicon and nitrogen in the areas while oxygen element is deficient. Figure 8(k) shows that a pyrolyzed component without cracks or millimeter-scale pores is achievable, when based on the addition of fillers, carefully designed heating rate and dwelling temperature are employed, so that the structural changes are allowed to evolve in a slow pace and released gases have sufficient time to escape.

The filler-induced pores observed in Figure 8(g) are yet present in this sample as can be seen in Figure 8(l); however, the number of these defects reduced remarkably in comparison with Figure 8(g) hinting structural improvement at macroscopic as well as microscopic scale. As the two specimens possess same composition and comparable microstructure, this structural improvement is solely ascribed to the processing conditions, namely the heating profiles during pyrolysis. Thus, employing a slower heating rate at temperatures of high gas release allowed the fine pores in the microstructure to sufficiently decrease the gas buildup inside the material as observed previously [51]. This in turn decreased the shape deformations and porosity leading to successful printing of the PDC objects. This slow ceramization process along with performance gain by the addition of filler particles resulted in improved mechanical properties of fabricated parts. The reduced shape deformations achieved by the customized heating profile are in agreement with the statement of fracture occurring at high heating rates with high possibility during pyrolysis-induced gas evolution [26]. The final steps of gas release, which are responsible for the ending of viscous flow in material, would cause hardening with a gradient between the exterior and interior leading to microcracks.

**Figure 9.**
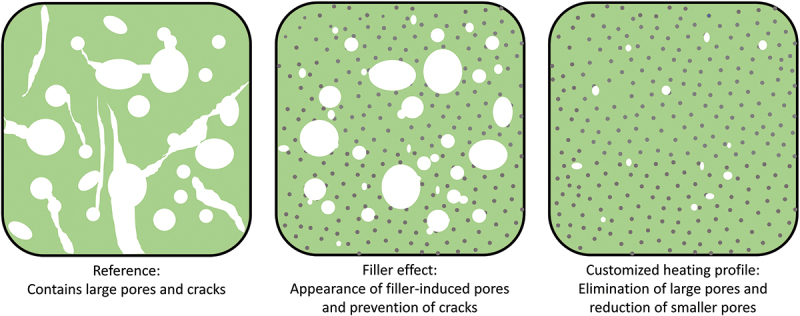
Representative microstructure images of pyrolyzed resins showing the structural changes due to filler addition and employing a customized heating profile during pyrolysis with (left) preceramic polymer resin without fillers, (middle) preceramic polymer resin with 10 vol.% filler concentration, (right) preceramic polymer resin with 10 vol.% filler concentration undergoing customized pyrolysis profile.

Summarizing, the most significant advantage of including the Si_3_N_4_ particles in resin was the evasion of crack formation, whereas extending the time allowed for the thermally induced structural transformation efficiently prevented generation of larger pores within material. Based on the characterization results in this work, the following representation of the microstructural changes of the pyrolyzed material was generated (Figure 9). Here, the ceramic obtained from reference resin (Resin 1) is shown with large balloon-like pores and cracks across the structure. Thus, the addition of the Si_3_N_4_ particles improves the shape retention by reinforcing the material as well as preventing cracks via gas escape channels. And finally, the extended time allowes key structural transformation periods during pyrolysis further contributes to the generation of additively manufactured PDC objects with minimal defects.

The most successful sample from this work, which is the initially 10 vol.% loaded (final particle load calculated as ~40.6 wt.%) sample obtained with the customized pyrolysis profile, had 24% linear shrinkage and 35.5% mass loss without shape deformation during the pyrolysis. These values can be further improved with a higher filler loading as linear shrinkage, porosity and weight loss can be reduced with increased filler concentration [37]. Moreover, an increase in strength is expected because of the reinforcing filler presence as well as the decreasing pore volume. However, for very high filler loading (>40 vol.%), it is possible to lose the some of the additional strength due to excessive pores resulting from isolated resin regions [30]. Furthermore, we were able to demonstrate that objects with 2 mm wall thickness are possible to be prepared with our approach as opposed to the prevalent claims in the literature, which is well above the 0.5 mm ‘safe limit’.

## Conclusions

The Si_3_N_4_-filled polyvinylsilazane resins were additively manufactured with a DLP printer through careful optimization of resin composition and printing parameters. The photopolymerization was realized at a much lower photoinitiator concentration (0.5 wt.% of the liquid phase of resin) compared to other cerSLA studies and the viscosity of the resin was reasonably low. Moreover, the cure depth lowering effect of dispersants by breaking agglomerations was demonstrated. Furthermore, it was found that using glass as build stage material increased adhesion with the chosen material system and can be helpful for similar setups. Among the pyrolyzed 3D printed objects, reference PVSZ-derived ceramic demonstrated the highest mass loss (47.4%) and linear shrinkage (35%), while adding 10 vol.% Si_3_N_4_ combined with the customized pyrolysis profile could reduce them to 35.5% and 24%, respectively. Moreover, the skeletal density of the 2 mm sample without fillers was 2.26 g/cm^3^, which changed to 2.42 g/cm^3^. This sample was the only sample, which retained its initial shape during shrinking. In addition, the effect of fillers incorporation and custom pyrolysis profile on the resultant microstructure is demonstrated. The filler effect on the PDC matrix was observed by a significant reduction of cracks and macroscopic pores owing to the formation of filler-induced pores and thereby diffusion channels for the emitted gas. Subsequently, the effect of custom heating resulted with the complete elimination of residual cracks and millimeter-scale pores besides the reduction in filler-induced pores. This underlined the necessity of employing slower heating rates during periods of intense structural change. Also, the pyrolysis duration was shortened with this method compared to the pyrolysis studies done with a single and slow heating rate. Finally, the thicker samples showed severe shape deformations; however, the samples without fillers of all thicknesses demonstrated the worst shape deformations. A higher filler loading is possible and may allow the production of bulkier PDCs without shape deformations or cracks, but further optimization is required to prevent the exposure time being impractically long. Further developments of strategies to overcome the deformation and fracture of PDCs during pyrolysis can lead to high temperature resistant, highly chemically inert products with elevated strength. Exemplary applications of such additively manufactured PDCs can be thermal protection systems, electronic device packaging, high-temperature heat exchangers and microreactors.

## Supplementary Material

Supplemental Material
