# Supplementary material for: Influence of Si3N4 fillers and pyrolysis profile on the microstructure of additively manufactured silicon carbonitride ceramics derived from polyvinylsilazane
Source: Sci Technol Adv Mater. 2024 Jun 6;25(1):2363170. doi: 10.1080/14686996.2024.2363170 (PMC11188956; doi:10.1080/14686996.2024.2363170)
Supplement: Supplemental Material [file TSTA_A_2363170_SM0578.docx]

**Supplemental Information**

**Influence of Si_3_N_4_ Fillers and Pyrolysis Profile on the Microstructure of Additively Manufactured Silicon Carbonitride Ceramics Derived from Polyvinylsilazane**

Alper Balkan^†^*, Xifan Wang, Aleksander Gurlo*

Technische Universität Berlin, Faculty III Process Sciences, Institute of Materials Science and Technology, Chair of Advanced Ceramic Materials, Straße des 17. Juni 135, 10623 Berlin, Germany

^†^Present address: Laboratory for Processing of Advanced Composites (LPAC), École Polytechnique Fédérale de Lausanne (EPFL), CH-1015 Lausanne, Switzerland

*Corresponding authors, alper.balkan@epfl.ch, gurlo@ceramics.tu-berlin.de

**Photo-curing behavior of resins with and without Si_3_N_4_**

Table S1. Cure depth measurement results of resins.

| **Measurement Series Name** | **Exposure Time (s)** | | | | | | | |
| --- | --- | --- | --- | --- | --- | --- | --- | --- |
|  | 2 | 3 | 4 | 5 | 10 | 20 | 30 | 300 |
|  | **Cure Depth (μm)** | | | | | | | |
| Without Si_3_N_4_ |  |  |  |  | 1080 | 1860 | 2390 |  |
| Grey Si_3_N_4_ – 1 | 40* |  | 50 | 50 | 80 | 80 | 210 | 240 |
| Grey Si_3_N_4_ – 2 |  |  |  | 42* | 46* | 54* | 47 | 100 |
| White Si_3_N_4_ |  | 80 | 80 | 90 | 100 | 120 | 210 | 320 |

*: Film has wrinkled texture due to naturally occurred mini-foldings, causing inflated measured thickness.


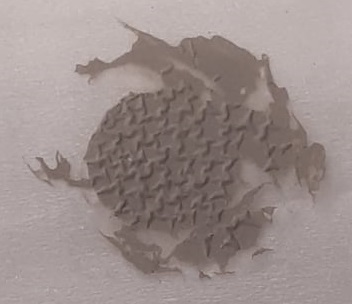


Figure S1. Image of the Grey Si_3_N_4_ – 3 sample with wrinkled texture after 2 s exposure.

**Particle and dispersant effects on resin viscosities**


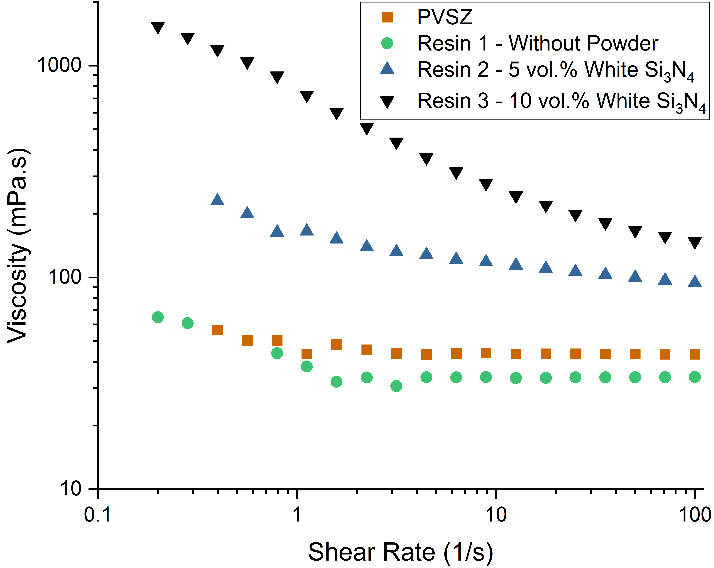


Figure S2. The dynamic viscosity measurement values of PVSZ and Resins 1-3.

Table S2. The viscosities of samples at 1.12 s^-1^ shear rate.

| **Sample Name** | **Dispersant Amount (wt.%)*** | **Powder** | **Powder Amount (vol.%)**** | **Viscosity at 1.12 1/s Shear Rate (mPa.s)** |
| --- | --- | --- | --- | --- |
| PVSZ |  |  |  | 43.55 |
| Resin 1 |  |  |  | 37.98 |
| Resin 2 | 5 | White Si_3_N_4_ | 5 | 165.25 |
| Resin 3 | 5 | White Si_3_N_4_ | 10 | 727.19 |

* wt.% with respect to the total weight of the liquid phase of resin

** vol.% with respect to the resin

**Composition of evolved gases in mass spectrometry**

Table S3: The assigned species of gases and their respective peaks.

| **m/z** | **Composition** | **Peaks (°C)** |
| --- | --- | --- |
| 2 | H_2_ | 291, 566, 776 |
| 12 | CH_4_ | 291, 561 |
| 13 | CH_4_ | 291, 561 |
| 14 | CH_4_, NH_3_ |  |
| 15 | CH_4_, NH_3_ | 296, 566 |
| 16 | CH_4_, NH_3_, H_2_O | 291, 566 |
| 17 | NH_3_, H_2_O | 291 |
| 18 | H_2_O | 296 |
| 28 | SiH_4_, C_2_H_4_ |  |
| 32 | SH_2_, SiH_4_, O_2_ |  |
| 34 | SH_2_ | 296 |
| 42 | SiNH_5_, SiCH_6_, C_3_H_6_ | 296 |
| 44 | CH_3_SH, CO_2_, SiO, SiNH_5_, SiCH_6_ | 291, 361, 536 |
| 46 | CH_3_SH, SiNH_5_, SiCH_6_ | 301 |
| 47 | CH_3_SH, SiNH_5_ | 296 |
| 48 | CH_3_SH, C_4_H_8_, SO | 301,361 |
| 56 | C_4_H_8_ | 296, 496 |
| 59 | Other silicon-containing compunds | 301 |
| 64 | SO_2_, other silicon-containing compunds | 301, 371 |
| 73 | Other silicon-containing compunds | 301 |

**Phases of Si_3_N_4_ in samples following pyrolysis**


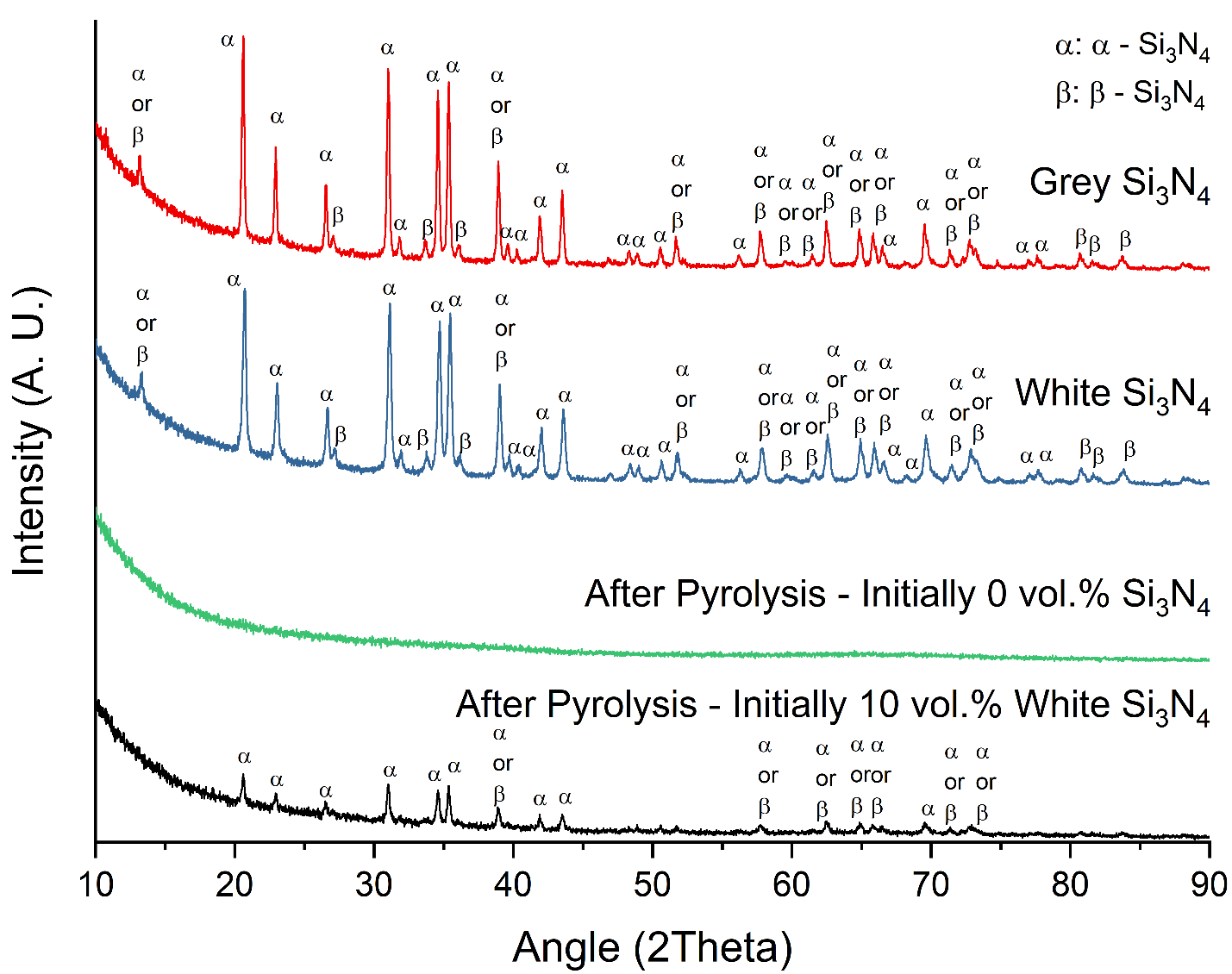


Figure S3. X-ray diffraction measurements of raw Si_3_N_4_ powders and PDCs with the peaks assigned to their respective material phases based on [1,2].

[1] J.W. Reutenauer, T.P. Coons, C.L. Hill, K.A. Arpin, M.A. Kmetz, S.L. Suib, Synthesis and characterization of polyvinylsilazane as a precursor for Si3N4 based ceramic materials, J Mater Sci. 46 (2011) 6538–6544. https://doi.org/10.1007/s10853-011-5600-y.

[2] C.R. Blanchard, S.T. Schwab, X-ray Diffraction Analysis of the Pyrolytic Conversion of Perhydropolysilazane into Silicon Nitride, Journal of the American Ceramic Society. 77 (1994) 1729–1739. https://doi.org/10.1111/j.1151-2916.1994.tb07043.x.

**Elemental constituents within material originated from the pyrolyzed polyvinylsilazane resin**


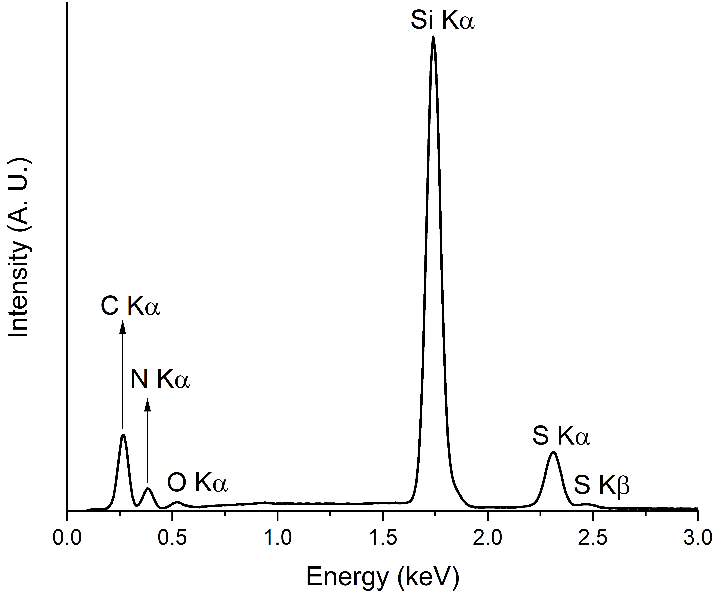


Figure S4. EDX emission spectrum of Sample 1.
